# Pregnancy outcomes in women with liver disease: Is pregnancy safe? A cross-sectional study

**DOI:** 10.18502/ijrm.v13i10.7774

**Published:** 2020-10-13

**Authors:** Reza Shekarriz-Foumani, Fakhrolmolouk Yassaee, Sara Tarokh, Mahbobeh Taheri

**Affiliations:** ^1^Department of Community Medicine, School of Medicine, Shahid Beheshti University of Medical Sciences, Tehran, Iran.; ^2^Department of Obstetrics and Gynecology, Genomic Research Center, Taleghani Hospital, Shahid Beheshti University of Medical Sciences, Tehran, Iran.; ^3^Taleghani Hospital, Shahid Beheshti University of Medical Sciences, Tehran, Iran.

**Keywords:** Liver diseases, Maternal, Outcome, Neonatal, Pregnancy.

## Abstract

**Background:**

There is evidence suggesting that the pregnancy outcome may be affected by some medical conditions, such as liver diseases.

**Objective:**

The present study aimed to investigate the prevalence of liver disease and its outcomes in pregnant women referred to antenatal clinic in the hospital.

**Materials and Methods:**

In this cross-sectional study, all pregnant women with abnormal liver function test attending antenatal clinic affiliated to Shahid Beheshti University of Medical Sciences were recruited from August 2017 to July 2018. All participants were followed-up until delivery with respect to the maternal and neonatal outcome.

**Results:**

Of a total of 7,121 pregnant women recruited in the study, 110 (1.58%) women were detected with a liver disease; of these, 105 women were diagnosed with pregnancy-specific liver diseases, including HELLP syndrome (10.9%), preeclampsia (50.98%), partial HELLP (0.9%), eclampsia (0.9%), acute fatty liver (9.1%), intra-hepatic cholestasis 25 (22.7%), and 5 women the non-pregnancy-specific liver disease, including Liver transplantation (2.7%), and Autoimmune hepatitis (1.8%). Prevalence of the premature birth was 64.5% in pregnancy-specific liver disease, but no premature birth was detected in cases with liver transplantation. We found that neonatal mortality was significantly associated with neonatal prematurity (p = 0.013), IUGR (p < 0.001), placental pathology (p = 0.04), we had no maternal mortality.

**Conclusion:**

Liver disease is not uncommon in pregnancy. This study demonstrated that pregnancy is safe in women with liver disease.

## 1. Introduction

About 3-5% of pregnancies are complicated by an abnormal liver function test, which can affect pregnancy outcomes depending on the severity of the disease. Several factors may be resulted in abnormal liver enzyme tests in pregnant women, including physiological changes in pregnancy, acquired the liver disease during pregnancy, pre-existing liver disease, and pregnancy-related liver disease. Interestingly, an abnormal liver function test, which results due to the physiologic changes and occurs in pregnant women without liver dysfunction, had a unique pattern and can be recognized when compared with the normal range of liver function test (1).

Preeclampsia is considered as the most common cause of liver dysfunction in pregnancy. Some cases are further complicated by HELLP, partial HELLP syndrome [H (hemolysis), EL (elevated liver enzymes), LP (low platelet count)], and eclampsia. Delivery is the only definitive therapy in such conditions, since many maternal complications may occur, including placental abruption, renal failure, subcapsular hematomas, and hepatic rupture (2-6). Cholestasis of pregnancy, which is typically presented by generalized pruritus, predominately on the palms of the hands and the soles of the feet, and the condition is worse at night, may be associated with liver dysfunction (7-14). Acute fatty liver of pregnancy is a sudden fatal disorder occurring almost exclusively in the third trimester of pregnancy; fatty infiltration of hepatocytes causes acute hepatic failure with coagulopathy and encephalopathy. Due to this fatal liver dyfunction, early diagnosis and immediate delivery is essential for maternal and fetal survival (15). Also, liver transplantation is considered to be the choice treatment for end-stage liver disease and its success has led to an increase in the number of female liver transplantation. While reproductive ages women with autoimmune hepatitis may become pregnant, the pregnancy outcome has not well clarified in such women. On the other hand, the complications of liver disease in pregnancy can affect the maternal and neonatal health, although there is no comprehensive study investigating these outcomes in Iranian pregnant women. Therefore, this study aimed to investigate the prevalence of liver disease and evaluate pregnancy outcomes in these patients in the population of pregnant women referred to prenatal clinics affiliated to Shahid Beheshti University of Medical Sciences (SBMU), Tehran, Iran.

## 2. Materials and Methods

In this cross-sectional study, of 7121 pregnant women attending to anetatal clinic affiliated to SBMU assessed for eligibility critera, the number of 110 pregnant women with various types of liver disease were recruited from August 2017 to July 2018. Our inclusion criteria were all pregnant women with liver disorders during pregnancy, those with previous hepatic disorders and/ or those with previous liver transplantation. Women with drug induced abnormal liver function test were excluded from the study.

The diagnosis of liver disease was made based on the symptom and sign and liver function tests including SGOT, SGPT, Bilirubin were studied along with some more definitive tests such as CBC, Plasma glucose, Urinary protein, Urea, Creatinine, LDH, Peripheral smear to aid identification of underlying cause. Study participants were admitted in the obstetric ward. After obtaining the demographic, menstrual and obstetric histories, the specific symptoms related to liver dysfunction such as pruritus, persistant vomiting, blurring of vision, diminished urine output,upper abdominal pain and anorexia were asked, and then a thorough general and obstetric examination were carried out for all participants. During hospitalization, all clinical manifestation were observed and laboratory test was checked two times per week. Any deterioration in clinical manifestation, such as severe hypertension, severe headache, epigastric pain, or change in laboratory test, such as increasing liver enzymes or creatinine, termination of pregnancy was done. Patients were followed up till two weeks postpartum.

HELLP syndrome was diagnosed based on the following criteria: complete: raised bilirubin, elevated AST (> 70 IU/L), low platelet count (< 100,000/μL), hemolysis (suggestive peripheral smear with schiztocyte along with increased reticulocytes). Partial: elevated AST (> 40 IU/L), low platelet count (< 150,000/μL), the absence or presence of hemolysis.Pre-eclampsia-associated liver dysfunction was diagnosed based on the elevated transaminases or bilirubin in the presence of hypertension to the extent of 140/90 mmHg or more on two occasions > 6 h apart, proteinuria (1+) after 20 weeks of pregnancy.Intrahepatic cholestasis of pregnancy (ICP) was detected based on having pruritus without any skin problem or allergy with elevated transaminases. Relieved after delivery. Acute Fatty Liver of Pregnancy (AFLP) was diagnosed with having six or more signs, including-vomiting, abdominal pain, encephalopathy, leucocytosis, elevated bilirubin, elevated transaminases, marked hypoglycaemia, renal impairment, coagulopathy.Autoimmune hepatitis was diagnosed for patients with chronic hepatitis, and increased liver enzymes.

All maternal information, including liver enzyme reversibility to the normal range, mode of delivery, maternal age, gestational age, and neonatal information, including Apgar score, birth weight, prematurity, intrauterine growth restriction (IUGR), and neonatal mortality were extracted from the postpartum records of the participants.

### Ethical consideration

This study was approved by the ethics committee of the Shahid Beheshti University of Medical Sciences (IR.SBMU.MSP.REC.1398.917) and informed consent was obtained from all participants.

### Statistical analysis 

Data were analyzed using the SPSS software version 21 (Statistical Package for the Social Sciences,version 21.0, SPSS Inc, Chicago, Illinois, USA). The Chi-square Fisher and U-Mann-Whitney tests were used to compare the study parameters. P < 0.05 was considered significant.

## 3. Results

Of a total of 7121 pregnant women referred to our clinic, 110 (1.58%) cases were detected with a liver disease with an age range of 16-49 yr (31 ± 6 yr); of these 105 women were diagnosed with pregnancy-specific liver diseases, including HELLP syndrome (10.9%) preeclampsia (50.98%), partial HELLP (0.9%), eclampsia (0.9%), acute fatty liver (9.1%), and intra-hepatic cholestasis 25 (22.7%) and 5 women were diagnosed with non-pregnancy-specific liver disease, including liver transplantation (2.7%), and autoimmune hepatitis (1.8%). The mean gestational age was 35 wk, and premature birth was 64.5%. In the participants with liver transplantation, the mean age of pregnant women was 29 years and the mean gestational age was 37 weeks. The mean neonatal weight was 2,696 gr and the mean apgar score was 8. Cesarean section was indicated for all cases due to physicians' requests. No maternal mortality, IUGR, and premature neonates were detected among participants studied. Table I shows the relationship between the participants' characteristics and neonatal mortality. The neonatal mortality was detected in preterm neonates with gestational age 27.6 (5.16) wk, but not in the neonates with gestational age 35.6 (2.9) wk (Table I). There was a statistical significant relationship between neonatal prematurity (p = 0.013), gestational age (p < 0.001), IUGR (p < 0.001), placental pathology (p = 0.041), neonatal weight (p = 0.001) with neonatal mortality in this study (Table I).

**Table 1:** The relationship between participant's characteristics, gestational age, neonatal weight, Apgar score with neonatal mortality


**Variables**		**Neonatal mortality**		**Total**	P-value*
	**Yes**	**No**	
**History of autoimmune disease** ${}^{*}$					
	**Yes**	1 (10)	4 (4)	5 (4.5)	0.385
	**No**	9 (90)	96 (96)	105 (95.5)	
**Liver function test returning to normal after delivery** ${}^{*}$					
	**Yes**	8 (80)	88 (88)	96 (87.3)	0.613
	**No**	2 (20)	12 (12)	14 (12.7)	
**Neonatal prematurity** ${}^{*}$					
	**Yes**	10 (100)	61 (61)	71 (64.5)	0.013
	**No**	0 (0)	39 (39)	39 (35.5)	
Intra-uterine growth restriction${}^{*}$					
	**Yes**	10 (100)	5 (5)	15 (13.6)	< 0.001
	**No**	0 (0)	95 (95)	95 (86.4)	
**Placental pathology** ${}^{*}$					
	**Normal**	8 (80)	98 (98)	106 (96.4)	0.041
	**Abnormal**	2 (20)	2 (2)	4 (3.6)	
**Presentation** ${}^{*}$					
	**Cephalic**	7 (70)	90 (90)	97 (88.2)	0.095
	**Breech**	3 (30)	10 (10)	13 (11.8)	
**Delivery method** ${}^{*}$					
	**NVD**	0 (0)	19 (19)	19 (17.3)	0.137
	**Cesarean section**	10 (100)	81 (81)	91 (82.7)	
Gestational age${}^{**}$		193.4 ±36.124	249.11 ± 20.503	244.05 ± 27.357	<0.001
Neonatal weight${}^{**}$		1072.50 ± 672.28	2844.55 ± 642.37	2683.46 ± 808.26	<0.001
Apgar score${}^{**}$		5 ± 2.449	8.64 ± 0.659	8.31 ± 1.413	<0.001
Fisher exact test for qualitative & man whitney U Test for quantitative variables, ${}^{*}$ Data presented as n (%), ${}^{**}$ Data presented as Mean ± SD					

## 4. Discussion

In this study, we evaluated the pregnancy outcomes in pregnant women with liver disease. We found that in pregnant women with autoimmune hepatitis, the main outcome was a premature birth and no maternal mortality was detected in these patients. In contrast with our study findings, Orgul *et al.*, showed two cases of autoimmune hepatitis in which one maternal mortality was occurred (16). Nowadays, the overall perinatal mortality is decreased because of the better understanding of physiologic changes during the pregnancy, team working, and on-time intervention. In agreement with previous studies, the prevalence of the liver disease in pregnant women in our study was 1.58% (4, 8).

This study showed that the mean age of the pregnant women with liver disease was 31 ± 6 yr. Similarly to our study, Tiwari *et al*. assessed a young study population with aged range of 20 to 29 years.They believed that a variety of liver tests is very important before pregnancy for this age range, especially if there is a history of past medical diseases, such as chronic hypertension, diabetes mellitus, autoimmune hepatitis, and liver disease (4). In our study, 25.5% of our participants had a history of hypertension and 10.9% had previous liver disease.

The relationship between the gestational age at the time of delivery and neonatal prematurity, has been studied in several studies (1-15). The mean gestational age in our study was 244.05 ± 27.357 days, and the preterm birth was detected in 64.5% of women that is high and corresponds with previous studies (1-6, 8-15). In the study of Carballo *et al*., GA was 35 wk and 6 days with 10% premature delivery. The low prevalence of adverse fetal outcomes in their study could be related to the low rate of prematurity (7). According to the results of this study, we can estimate that the percentage of premature births in mothers with liver disease was obviously high and this needs the availability of medical equipment (such as NICU) at accessible sites of such patients. This study showed that the most common diseases related to liver dysfunction were preeclampsia/ eclampsia with a rate of 51.88% and cholestasis of pregnancy with a rate 22.7%. Likewise, Tiwari *et al*. found that preeclampsia and cholestasis were the most common complications related with liver diseases with a prevalence of 66.35% and 16.8%, respectively (4).

The present study showed that in the participants with liver transplantation, the mean maternal age was 29 years and the mean gestational age was 37 wk. The mean neonatal weight was 2,696 gr, and the mean Apgar score was 8. Cesarean section in all these cases was performed due to physicians' request. This finding is in contrast with the study by Costa and colleagues in which preterm delivery was 12.5-50% (17).

Accordingly, in overall, pregnancy outcomes in pregnant women with liver transplants can be considered satisfactory. Meanwhile, these results coincided with the findings of previous studies (17-19), which found that women with chronic liver disease are benefiting from excellent results after liver transplantation with proper healthcare. In line with previous studies, this study showed that the mean weight of the neonate was 2683 gr (5-7, 9-10). It is well documented that the neonatal prematurity causes a lack of maturation of the neonate's lung and oxygenation to the body and brain, which may cause neonatal brain retardation, the increased risk of mortality, and the need for medical equipment (such as NICU). Similarly to one study conducted by Carballo *et al*. (7), our study results shows that the mean neonatal Apgar score is 8.31 ± 1.41, which is in the normal range.

Consistent with earlier studies (1, 5, 6, 17-19), in our study, a large number of deliveries (82.7%) were performed by cesarean section, which is a high rate. On the contrary, Rook *et al*. reported different results in this regard. In their study, 84% of women were delivered through normal vaginal delivery (12). Due to the adverse effects of cesarean section on the mother and the baby, diagnosis, treatment, and care of the liver can minimize this mode of delivery. Our study showed that hepatic enzymes were returned to normal after delivery in 87.3% of cases within 2 wk.

The neonatal mortality in this study was 9.1%. The rate of neonatal mortality in developing countries such as India was reported as 29.17% (4). We found no maternal mortality due to liver diseases. Since the maternal mortality is of the most important and crucial health indicator related to the pregnancy, this finding is considered satisfactory and indicating that liver disease is not a serious threat to the mother. We had no cases of any type of viral hepatitis, in contrast to the study by Krishnamoorthy *et al*. who reported pregnant women with viral hepatitis and high perinatal mortality (20). The main limitation of our study was the small number of cases such as liver transplantation.

## 5. Conclusion

This study demonstrated that the liver disease in pregnancy is not a risk to the mother and most mothers return to normal after giving birth and can take care of their newborns. However, timely diagnosis, intervention, and monitoring of mother and fetus with the teamwork of obstetricians, neonatologists, gastroenterologists and anesthetists can prevent and reduce fetomaternal morbidity and mortality.

##  Conflict of Interest 

The authors declare that there is no conflict of interest.

## References

[1] Pan C, Perumalswami PV. Pregnancy-related liver diseases. *Clin Liver Dis* 2011; 15: 199–208. 10.1016/j.cld.2010.09.007 21112001

[2] Solanke D, Rathi C, Pandey V, Pati M, Phadke A, Sawant P. Etiology, clinical profile, and outcome of liver in pregnancy with predictors of maternal mortality: A prospective study from western india. *Indian J Gastroenterol* 2016; 35: 450–458. 10.1007/s12664-016-0704-6 27796940

[3] Karegoudar D, Dhirubhai PR, Dhital M, Amgain K. A study of liver disorder and its consequences in pregnant women with jaundice in tertiary care centre in Belgaum, Karnataka, India. *Journal of Dental and Medical Sciences* 2014; 13: 14–18.

[4] Tiwari A, Aditya V, Srivastava R, Gupta G. A study of spectrum and outcome of liver diseases in pregnant women at BRD medical college. *Int J Reprod Contracept Obstet Gynecol* 2017; 6: 3641–3645.

[5] Esposti SD. Pregnancy in patients with advanced chronic liver disease. *Clin Liver Dis* 2014; 4: 62–68. 10.1002/cld.415 PMC6448737 30992923

[6] Rezk M, Gamal A, Emara M. Maternal and fetal outcome in de novo preeclampsia in comparison to superimposed preeclampsia: a two-year observational study. *Hypertens Pregnancy* 2015; 34: 137–144. 10.3109/10641955.2014.982329 25548836

[7] Carballo-Nunez E, Gonzalez-Rodrlguez L, Gonzalez-Boubeta R, Maria Teresa AP. Perinatal outcomes in patients with cholestasis of pregnancy. *Ginecol Obstet Mex* 2015; 83: 776–784. 27290802

[8] Bacq Y, Sentilhes L. Intrahepatic cholestasis of pregnancy: Diagnosis and management. *Clin Liver Dis* 2014; 4: 58– 61. 10.1002/cld.398 PMC6448735 30992922

[9] Bouwers L, Koster MPH, Page-christiaens GCML, Kemperman H, Boon J, Evers IM, et al. Intrahepatic cholestasis of pregnancy: maternal and fetal outcomes associated with elevated bile acid levels. *Am J Obstet Gynecol* 2015; 212: 100: e1–e7. 10.1016/j.ajog.2014.07.026 25046809

[10] Kurt A, Ecevit A, Kisa B, Anuk Ince D, Tarcan A, Bilgin Yanık F. Neonatal outcomes of pregnancy with intrahepatic cholestasis. *Perinatal Journal* 2011; 19: 10–14.

[11] Lin J, Gu W, Hou Y. Diagnosis and prognosis of early-onset intrahepatic cholestasis of pregnancy: a prospective study. *J Matern Fetal Neonatal Med* 2019; 32: 997–1003. 10.1080/14767058.2017.1397124 29065754

[12] Rook M, Vargas J, Caughey A, Bacchetti P, Rosenthal P, Bull L. Fetal outcomes in pregnancies complicated by intrahepatic cholestasis of pregnancy in a Northern California cohort. *PLoS One* 2012; 7: e28343: 1–6. 10.1371/journal.pone.0028343 PMC3293870 22403605

[13] Sharma P, Sarkar B, Majhi B. Fetal and neonatal outcomes in intrahepatic cholestasis of pregnancy. *Int J Reprod Contracept Obstet Gynecol* 2018; 7: 4056–4060.

[14] Wikstrom Shemer E, Marschall HU, Ludvigsson JF, Stephansson O. Intrahepatic cholestasis of pregnancy and associated adverse pregnancy and fetal outcomes: a 12-year population-based cohort study. *BJOG* 2013; 120: 717–723. 10.1111/1471-0528.12174 23418899

[15] Holub K, Camune B. Caring for the woman with acute fatty liver of pregnancy. *J Perinat Neonatal Nurs* 2015; 29: 32–40. 10.1097/JPN.0000000000000076 25633398

[16] Orgul G, Ozcan EU, Celik HT, Beksac MS. Autoimmune hepatitis and pregnancy: report of two cases with different maternal outcomes. *Clin Exp Hepatol* 2017; 3: 212–214. 10.5114/ceh.2017.71445 PMC5731429 29255809

[17] Costa MLB, Surita FGC, Passini Jr R, Cecatti JG, Boin IFS. Pregnancy outcome in female liver transplant recipients. *Transplant Proc* 2011; 43: 1337–1339. 10.1016/j.transproceed.2011.02.026 21620123

[18] Parhar KS, Gibson PS, Coffin CS. Pregnancy following liver transplantation: Review of outcomes and recommendations for management. *Can J Gastroenterol* 2012; 26: 621–626. 10.1155/2012/137129 PMC3441170 22993734

[19] Lindenmeyer CC, Mccullough AJ. Pregnancy outcomes and reproductive health after liver transplantation. *Clin Liver Dis* 2016; 6: 142–144. 10.1002/cld.518 PMC6490671 31041012

[20] Krishnamoorthy J, Murugesan A. Jaundice during pregnancy: maternal and fetal outcome. *Int J Reprod Contracept Obstet Gynecol* 2016; 5: 2541–2545.

